# Surface Acting or Deep Acting, Who Need More Effortful? A Study on Emotional Labor Using Functional Near-Infrared Spectroscopy

**DOI:** 10.3389/fnhum.2019.00151

**Published:** 2019-05-10

**Authors:** Yongbiao Lu, Wenfeng Wu, Gaoxing Mei, Shouying Zhao, Haibo Zhou, Daling Li, Deng Pan

**Affiliations:** ^1^School of Psychology, Guizhou Normal University, Guiyang, China; ^2^School of Education, Hunan University of Science and Technology, Xiangtan, China

**Keywords:** emotional labor, surface acting, deep acting, fNIRS (functional near-infrared spectroscopy), energy, prefrontal lobe

## Abstract

Emotional labor is characterized by two main regulation strategies: surface acting and deep acting. However, which strategy consumes more energy? To explore this, we used functional near-infrared spectroscopy (fNIRS) to measure changes in hemoglobin density while participants performed a task requiring them to make the opposite emotional facial expression of that presented in a picture. We found that (1) neither surface nor deep acting led to a significant change in hemoglobin concentration in the prefrontal cortex; (2) making negative and positive facial expressions activated the same left front and middle areas of the prefrontal cortex; and (3) making positive facial expressions activated the rear portion of the prefrontal cortex, but making negative facial expressions did not. Based on these findings and past work, we can infer that deep and surface acting may not significantly differ in terms of the activity in the prefrontal cortex energy consumed. Furthermore, engaging in positive and negative emotional labor appear to utilize some of the same neurological mechanisms, although they differ in others.

## Introduction

Emotional labor is the act of regulating one’s emotion to conform to organizational standards. Presently, it is central to numerous service occupations that employees are the first point of contact that customers have with the organization. Scholars have been continually seeking to understand the emotional labor process. Some have proposed that emotional labor consists of three components (Grandey and Gabriel, 2015): emotional requirement, emotional regulation, and emotional performance. The emotional requirements typically involve the “integrative” goal of showing positive displays and hiding negative ones [although, for certain occupations (e.g., teachers), these emotional requirements may involve negative or neutral displays; Schutz and Lee, 2014]. Emotional regulation refers to the effort expended by employees to comply with the socioemotional demands of the job. Researchers have pointed out three essential strategies of emotional regulation: surface acting, deep acting, and genuine emotional labor. Surface acting involves faking the required emotions: that is, when people engage in surface acting, they do not actually try to feel the emotions they wish to portray. They may put on “fake smiles” or other required emotional displays that do not reflect their true feelings. By contrast, deep acting involves putting effort into actually feeling and expressing the required emotions. When engaged in deep acting, people attempt to modify feelings to match the required display rules. Ashforth and Humphrey (1993) argue that, in addition to surface acting and deep acting, that there is a third form of emotional labor: spontaneous and genuine emotional labor, wherein the person expresses naturally felt emotions that align with the emotional display rules; in this case, no acting is required (Diefendorff et al., 2005). Emotional labor research has primarily focused on surface acting and deep acting (Grandey et al., 2013; Gabriel and Diefendorff, 2015). Finally, emotional performance means observable expressions of emotional labor. Pugh (2001) found that emotional performance was unrelated to employees’ reported moods, supporting the idea that these displays were strategic rather than actual expressions of feeling.

Since Hochschild (1983) coined the term “emotional labor,” scholars have designed numerous studies to explore its characteristics and mechanisms (e.g., Grandey, 2000). Among them, the different strategies of emotional labor are one of the most studied aspects—especially surface acting and deep acting (Grandey et al., 2013; Xanthopoulou et al., 2018). Meta-analyses have revealed that deep and surface acting have different effects (Hülsheger and Schewe, 2011; Mesmer-Magnus et al., 2012), and some interesting questions about these two strategies still remain to be answered. For example, what are the cognitive and social differences between these two strategies? Do they consume identical amounts of psychological resources or does one consume more? Most scholars believe that deep acting is more psychologically taxing than surface acting is. However, until now, there has been no real evidence to support this assertion. The most common way of examining these two emotion regulation strategies has been to ask employees to self-report the extent to which they use each strategy, and then examine the relationships between each strategy and various antecedents and outcomes (Gabriel et al., 2015). However, self-report measures often confound acting with affective motivational experiences; this is true for both surface acting (i.e., confounded with negative affect/stress) and deep acting (i.e., confounded with motivation). Furthermore, self-reported results might simply be reflecting effects of participants’ memory, and not emotional labor *per se*.

For this reason, some researchers have tried experimental methods to explore differences between surface acting and deep acting. For example, Shulei and Miner (2006) presented participants with differing sets of instructions, before they watched an emotion-eliciting film, to induce surface acting and deep acting. The authors found that (1) the participants devoted significant effort and found it difficult to perform both surface and deep acting, but deep acting engaged more attention, and (2) both surface acting and deep acting led to decreased sadness, while deep acting led to a stronger physiological response (e.g., greater increase in heart rate). In another experimental study, Gabriel and Diefendorff (2015) utilized a call center simulation to examine how shifts in customer incivility influenced continuous measures of participants’ felt emotions, surface acting, deep acting, and vocal tone during a single interaction. They found that surface acting and deep acting were used simultaneously to manage emotional labor demands. Although these two studies both used experimental methods, they still relied on participants’ self-ratings of surface acting and deep acting; in other words, they could not measure the effort used for emotional labor directly. Thus, we began to wonder if there is a way of measuring the effort used for emotional labor directly. The answer may be “yes.”

According to the definition of emotional labor, which refers to one kind of emotional regulation, so for surface acting and deep acting, they can be seen as the two emotional regulation strategies (Grandey, 2000; Grandey and Melloy, 2017). Gross (1998) suggested that the emotional regulation strategies could be categorized as *antecedent-focused* and *response-focused*, which Grandey (2000) in turn connected to surface and deep acting; deep acting is considered a form of reappraisal, which is linked to antecedent-focused regulation, whereas surface acting is linked to suppression, which is a type of response-focused regulation. A large number of neuroimaging studies have been conducted to examine the neural functions underlying the cognitive control of emotion (Hare et al., 2005; Goldin et al., 2008; Etkin et al., 2015; Zilverstand et al., 2017; Vandekerckhove et al., 2018). Accordingly, neuroimaging technology could be useful for exploring the neural mechanisms of emotional labor. However, there are few neural studies on emotional labor—why? We infer that the most important reason is the limitations such studies impose on participants’ actions; participants would have to utilize various expressive actions so that emotional labor can be experimentally studied, but most neuroimaging equipment requires participants to remain as immobile as possible. In most cases, this would rule out the use of neuroimaging techniques for emotional labor. However, functional near-infrared spectroscopy (fNIRS) can be used with less bodily constraints than other imaging modalities (Scholkmann et al., 2012). In fact, some researchers have used fNIRS to explore cortical areas that are associated with spontaneous facial affective expressions, human emotions, somatosensory stimulation, and other functions (Masataka et al., 2015; Naseer and Hong, 2015; Sun et al., 2015; Hong et al., 2017). Therefore, it may be suitable for use in emotional labor. Moreover, it is silent (i.e., it has no operating sounds), making it somewhat more comfortable for humans (Masataka et al., 2015).

Zapf (2002) discussed surface acting and deep acting from the perspective of action theory. According to this theory, deep acting would partly involve conscious processes at the intellectual level of action regulation. In other words, the person actively tries to influence his or her inner feelings to bring them in line with the emotions required by the organization. By contrast, surface acting is more likely to be triggered at the level of flexible action patterns—this implies that it is partly a routine process, and does not necessarily involve conscious processes. For example, despite feeling nothing, a salesperson might automatically display a smile. Based on Zapf’s view of deep and surface acting, we hypothesized that people will need greater psychological resources when engaging in deep acting than when engaging in surface acting.

In this study, the purpose was to verify whether people would need more psychological resources when engaging in deep acting than when engaging in surface acting. To induce use of the different strategies, we used emotional facial expressions. Because emotional labor involves the display of positive and negative emotions, we presented participants with positive or negative facial expressions and asked them to make the opposite facial expression. This also enabled us to explore whether there was a difference in the effort for these strategies between positive and negative emotional expressions.

## Materials and Methods

### Ethics Statement

All research procedures were approved by the Ethical Committee of Guizhou Normal University, Guiyang, Guizhou province, China. All participants provided written informed consent after fully understanding the study.

### Participants

Twenty male and 20 female senior undergraduates were recruited from a university in Hunan province, China. All participants came from a teacher training specialty (e.g., education or psychology) and were healthy and right-handed. None of them had a history of psychiatric or neurological disorders. Participants were randomized to the surface acting or deep acting condition. In the deep acting condition, one male and one female participant’s fNIRS data were not correctly obtained, so the data were excluded. The average age of all participants was 21.5 ± 1.4 years.

### Instrument

We used a 24-fiber (38-channel) fNIRS system (SHIMADZU LABNIRS System, Kyoto, Japan) in this study. Semiconductor lasers with wavelengths of 780 nm, 805 nm, and 830 nm are employed as light sources; every emitter in the LABNIRS system emits the three wavelength light, and as well as every detector measures the absorbance for each of these wavelengths. fNIRS measures the amount of relative change from the initial value of oxygenated hemoglobin (OxyHb), deoxygenated hemoglobin (deOxyHb), and total hemoglobin (totalHb), using the near-infrared rays. The increase in OxyHb and the concomitant decrease in deOxyHb reflects an increase in local arteriolar vasodilatation, which increases local cerebral blood flow and cerebral blood volume, a mechanism known as neurovascular coupling. This produces a change in the amount of light absorbed by this tissue, which can be measured by near infrared spectroscopy systems (Ferrari and Quaresima, 2012). These measurements are used as surrogates of brain activation (Bulgarelli et al., 2018). The more activated, the more OxyHb increases, the more energy will be consumed. The adequate depth of NIR light penetration (almost one half of the source-detector distance) can be achieved using a source-detector distance around 3 cm (Dehghani et al., 2009). Compared with other brain imaging devices, fNIRS is minimally restrictive and can accurately record measurements even when individuals are moving, making it useful for studying brain activity under more “natural” conditions. It is also totally non-invasive.

**FIGURE 1:**
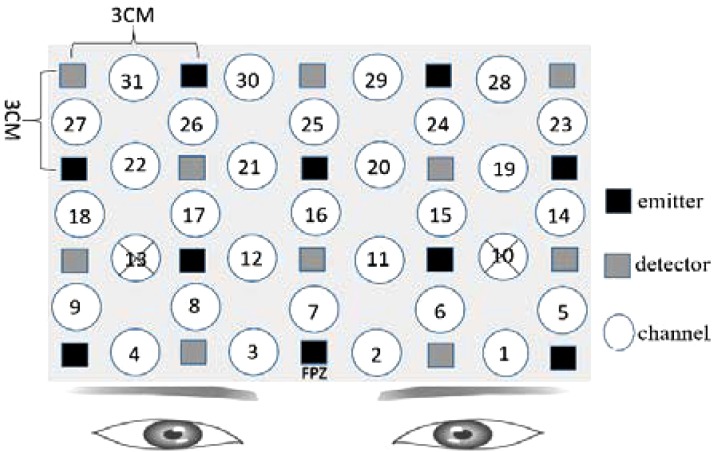
Functional near-infrared spectroscopy (fNIRS) settings. Ten emitters and 10 detectors were deployed with reference to the International 10–20 system, with a total of 31 channels. For channel 10 and channel 13, the distance of emitter and receptor was over 3 cm, so the measurement results were canceled. The “cross” mark was used to represent the canceled results.

Numerous recent studies have identified the prefrontal cortex (PFC) as a key region for the induction and regulation of emotional responses (Doi et al., 2013). As our fNIRS instruments could measure activity over a limited number of channels, we decided to affix the optodes of the fNIRS primarily on the PFC. Based on that, participants would make facial expression, and refer to a study by Hong et al. (2015). Therefore, we also placed some optodes on the pre-motor and supplementary motor cortexes. The details are shown in Figure 1. The optodes of fNIRS were fixed on a full head cap designed with reference to the International 10–20 system. The sampling rate was set to 30 Hz.

**FIGURE 2:**
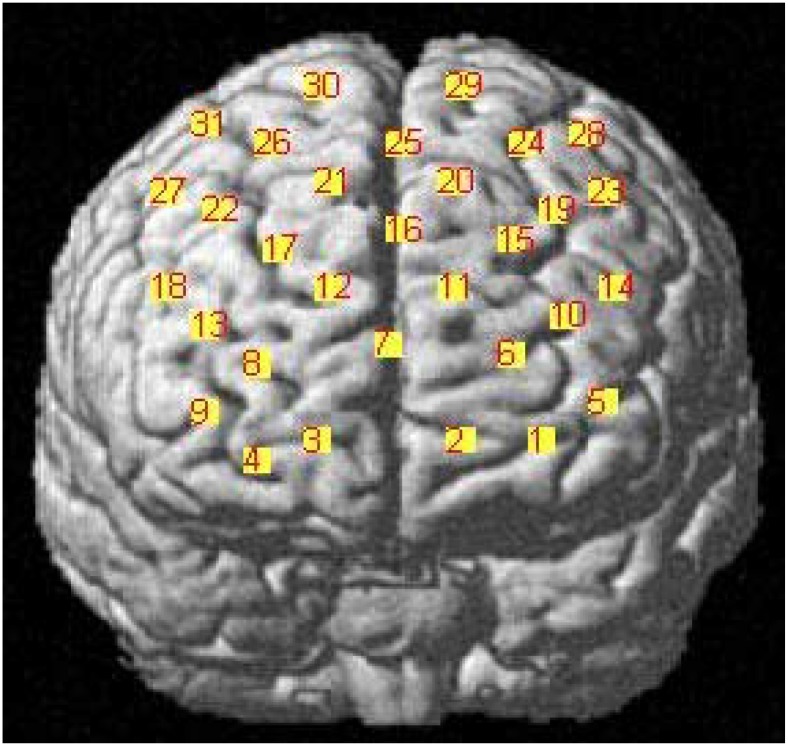
MNI locations for the channels.

We determined the anatomical locations of the optodes in relation to the standard head landmarks, including the nasion, Cz, left tragus (T3), and right tragus (T4), using a FASTTRAK 3D tracking system (Polhemus). The Montreal Neurological Institute (MNI) coordinates (Mazziotta et al., 2002) for the channels were obtained using the NIRS-SPM software (Singh et al., 2005; Ye et al., 2009) with MATLAB 2013b (Mathworks, Natick, MA, United States). The detailed MNI locations are shown in Figure 2 and in Table 1.

**Table 1:** Registered positions of fNIRS measurement channels on the standard brain MRI atlas.

Brodmann area	Channel
BA 6	29, 30, 31
BA 8	24, 25, 26, 28
BA 9	15, 16, 17, 19, 20, 21, 22, 23, 27
BA 10	2, 3, 5, 6, 7, 8, 9, 11, 12
BA 11	1, 4
BA 45	18
BA 46	10, 13, 14


*The channel numbers are defined in Figure 1, 2.*

### Materials

We selected 20 positive, 20 neutral, and 20 negative emotional face pictures from the Chinese Facial Affective Picture System (CFAPS; Bai et al., 2005) using CFAPS normative data. Among them, eight positive, eight neutral, and eight negative pictures were chosen to be used in the exercise phase, while the remaining were chosen to use in the formal phase. Emotion ratings were based on a nine-point rating scale. The average emotional valence rating of the selected pictures was 6.82 for the positive pictures, 4.32 for the neutral, and 2.34 for the negative. Half of all the pictures were of men, and the others were of women.

### Questionnaire

To identify whether participants had engaged with the tasks, we asked participants to complete a 4-item questionnaire after the experiment, as follows: (1) What did you think about the difficulty of the experiment? (2) How would rate the degree of effort put into the experiment? (3) How would you rate your degree of concentration on the experiment? (4) How would you rate your performance in executing the experimental instructions? Each item was rated on a six-point scale.

### Experimental Procedure

**FIGURE 3:**
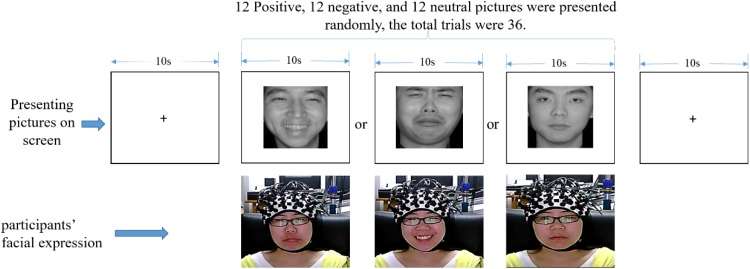
One trial experimental procedure (written informed consent was obtained for the publication of this image).

Participants were instructed to sit on a chair in front of a 17-in (32 × 24 cm) monitor. The distance between each participant and the monitor was set to around 70 cm. The fNIRS equipment was attached to participants’ heads. The experiment was divided into the exercise and formal experimental phases. In the exercise phase, participants practiced how to respond according to the instructions until they completely understood it. There were 24 facial expression pictures (eight positive, eight neutral, and eight negative), so in this phase, the participant would experience 24 trials under the set condition. Thereafter, the formal experiment began. Before the formal experiment, participants were instructed to avoid any head and body movements as much as possible while the fNIRS was operating. Subsequently, emotional face pictures were presented on the screen (315 × 356 pixels), and participants were asked to make the opposite facial expression of the pictures they viewed (see Figure 3 for details). In this phase, the participants would experience 36 trials (12 positive, 12 neutral, and 12 negative). The facial expression of the participants was video-monitored, in order to confirm that the correct facial expressions were displayed. To minimize the distraction of the participants, the experimental task was implemented in a dimly lit room.

### Surface Acting Condition

In this condition, we used the followed instructions to ensure that participants performed surface acting:

Welcome to the experiment!

First you will see a “+” sign in the middle of the screen; please look at it and keep your mind calm. After that, pictures of people with positive, negative, and neutral emotional expressions will be displayed on the screen. While focusing on the pictures, please adopt the facial expression opposite to the one presented in order to pretend to others that you are actually feeling that opposite emotion, and keep that expression until the picture disappears. If a neutral expression is displayed, please do not make any expression.

### Deep Acting Condition

As with the surface acting condition, we used instructions to induce participants to perform deep acting:

Welcome to the experiment!

First you will see a “+” sign in the middle of the screen; please look at it and keep your mind calm. After that, pictures of people with positive, negative, and neutral emotional expressions will be displayed on the screen. While focusing on the pictures, please adopt the facial expression opposite to the one presented, and try to actually experience the emotion you are expressing on your face. Please maintain that emotional state until the picture disappears. If a neutral expression is displayed, please do not make any expression.

### Data Preprocessing

As the distance between the emitting and detecting optodes was over 3 cm, channel 10 was interpolated by channels 1, 5, 6, 14, 15, and 19; and channel 13 was interpolated by channels 4, 8, 9, 17, 18, and 22. For this analysis, we chose to focus on changes in the concentration of OxyHb, as it is regarded as the most sensitive measure of changes in regional cerebral blood flow (Hoshi et al., 2001). Using the NIRS_SPM_V4_r1 toolbox (Ye et al., 2009), SPM8, and MATLAB 2013b (Mathworks, Natick, MA, United States), we processed participants’ individual data: (1) High-pass filtering: we selected a discrete cosine transform (DCT)-based on detrending algorithm. Based on DCT, the high-pass filtering was implemented by a specified cut-off of 40 s (frequency = 0.025 Hz) to remove unknown global trends due to breathing, cardiac events, vasomotion, or other experimental errors. These trends are the very low-frequency oscillations originating from slower changes in systemic cardiovascular properties, e.g., blood pressure (Gervain et al., 2011); (2) Low-pass filtering: according to the NIRS-SPM developer’s recommendation (Ye et al., 2009), we selected the precoloring method (Worsley and Friston, 1995) and the hemodynamic response function (*hrf*) to smooth the data. In this method, the intrinsic temporal correlations are swamped by an imposed temporal correlation structure, as smoothing the data with the temporal filtering will attenuate high frequency components; hence, this is a “low-pass filtering.” Since the transfer function of *hrf* is in the frequencies of modeled neuronal signals, cut-off frequency did not need to be set in this step; (3) Estimate: In the individual analysis, general linear model (GLM) parameters and temporal correlations were estimated, and beta values (average signal value of each channel) obtained under four conditions were calculated: condition 1 (a positive facial expression is presented); condition 2 (a negative facial expression is presented); condition 3 (a neutral facial expression is presented); and condition 4 (no facial expression, just a “+” sign is presented). Using these beta values, we conducted a group level analysis using SPSS Statistics 20.

## Results

### FNIRS Results

#### Activation of Channels

We compared the beta value of each channel with a 0 value. If the *p*-value was significant, and the *t*-value of the one simple *t*-test was positive, then the channel was defined to be activated; if the *t*-value was negative, the channel was defined to be deactivated; if the *p*-value was not significant, then the channel was defined to not be activated.

To determine which channels were activated while participants engaged in surface or deep acting, we subjected the beta values estimated from the NIRS-SPM tool to a one sample *t-*test and determined the level of activation when displaying positive, negative, and neutral emotional face pictures. The results are shown in Table 2. As shown in Table 2, according to the *BH_p* results, when participants viewed a positive facial expression (and therefore, made a negative expression), we found that no channel was activated for surface acting. For deep acting, channels 2, 9, 10, and 14 (BA10 and BA 46) were activated. When participants viewed a negative facial expression (and therefore, made a positive expression in response), those in the surface acting group showed activations in channels 14, 29, and 30 (BA 6 and BA 46); by contrast, those in the deep acting group showed no activated channels. When comparing surface acting and deep acting, the number of activated channels was slightly higher in the deep acting group than the surface acting group.

**Table 2:** Comparison of activated channels among different conditions.

	Surface acting (*n* = 20)						Neutral (*n* = 38)		Deep acting (*n* = 18)					
			
	Positive–negative			Negative–positive					Positive–negative			Negative–positive		
					
Channel	*t*	*P*	*BH_p*	*t*	*p*	*BH_p*	*t*	*p*	*t*	*p*	*BH_p*	*t*	*p*	*BH_p*
1	1.676	0.110	0.341	-0.398	0.695	0.798	-1.313	0.197	2.626	0.018^*^	0.110	1.521	0.147	0.301
2	-0.325	0.749	0.987	0.090	0.930	0.935	-0.719	0.477	3.348	0.004^**^	0.041^*^	-0.398	0.696	0.768
5	1.431	0.169	0.475	0.786	0.441	0.624	-0.841	0.406	1.639	0.120	0.265	2.336	0.032^*^	0.138
9	-0.22	0.982	0.987	1.275	0.218	0.562	-0.225	0.823	3.330	0.004^**^	0.041^*^	0.951	0.355	0.440
10	1.910	0.071	0.276	0.882	0.389	0.624	-0.578	0.567	3.139	0.006^**^	0.046^*^	2.416	0.027^*^	0.138
14	2.327	0.031	0.265	3.245	0.004^**^	0.044^*^	1.048	0.301	4.440	0.000^***^	0.011^*^	3.571	0.002^**^	0.073
15	1.083	0.292	0.620	0.992	0.334	0.624	0.897	0.376	2.255	0.038^*^	0.167	2.916	0.010^*^	0.133
18	1.766	0.093	0.322	-0.427	0.674	0.798	1.043	0.304	2.436	0.026^*^	0.135	0.367	0.718	0.768
23	-0.856	0.403	0.734	2.218	0.047^*^	0.272	1.158	0.254	1.386	0.184	0.380	2.777	0.013^*^	0.133
24	-2.283	0.034^*^	0.265	1.334	0.198	0.562	-0.181	0.857	-1.769	0.095	0.263	1.237	0.233	0.344
25	-2.011	0.059	0.266	2.247	0.037^*^	0.272	-0.571	0.571	-0.319	0.754	0.899	1.355	0.193	0.340
26	-2.615	0.017^*^	0.264	0.973	0.343	0.624	-0.162	0.872	0.060	0.953	0.992	0.977	0.342	0.440
28	-2.811	0.011^*^	0.264	1.918	0.070	0.272	0.951	0.348	-1.318	0.205	0.397	2.282	0.036^*^	0.138
29	-1.999	0.060	0.266	3.519	0.002^**^	0.035^*^	-0.659	0.514	-0.627	0.539	0.795	2.509	0.023^*^	0.138
30	-2.135	0.046^*^	0.266	4.306	0.000^***^	0.012^*^	-0.683	0.499	0.010	0.992	0.992	2.061	0.055	0.155
31	-1.370	0.187	0.482	1.963	0.064	0.272	0.579	0.566	-0.541	0.596	0.839	2.626	0.018^*^	0.137


*BH_p is the multiple comparison *p*-value correction using the Benjamini–Hochberg procedure; ^∗∗∗^*p* < 0.001; ^∗∗^*p* < 0.01; ^∗^*p* < 0.05. The asterisk marks annotation for the following tables are the same as this table. Only the channels with significant *p*-value are displayed in this table.*

#### Repeated Measures ANOVA

We then subjected the OxyHb data to a 2 × 3 repeated measures analysis of variance (rmANOVA). The independent variables were group and facial expressions. Group was a between-subject variable, and included two levels: surface acting and deep acting. Facial expression was a within-subject variable, and had three levels: positive, negative, and neutral valence pictures. The results indicated a non-significant interaction of the group and facial expressions for all channels (all *P* > 0.05), and a non-significant main effect of the group expression (all *P* > 0.05). However, the main effect of the facial expression was significant for some channels. We further merged the surface acting (*n* = 20) and deep acting group (*n* = 18) into one group (*n* = 38), and used facial expression as an independent variable to run a one-way repeated measures ANOVA. The results showed that facial expression was significant for some channels. For details, please see Table 3. Specifically, according to *BH_p* results, a significant effect of facial expression was found for channel 1 (located in left orbitofrontal area, BA11); channels 5 (located in the left frontopolar area, BA10); and channels 10, 14 [located in the left dorsolateral PFC (L-DLFPC), BA46, please see Figure 2]. In addition, in channels 24, 25, and 28 (located in the frontal eye fields, BA8) and channels 29, 30, and 31 (located in the pre-motor and supplementary motor cortexes, BA6), facial expression had a significant effect.

**Table 3:** Main effects of facial expression (*n* = 38).

Channel	*F*	*p*	*BH_p*	η^*2*^	PHMC	Channel	*F*	*p*	*BH_p*	η^*2*^	PHMC
1	6.473	0.004^**^	0.018^*^	0.27	a > c	17	0.071	0.931	0.931	0.004	None
2	0.861	0.431	0.534	0.047	None	18	2.693	0.082	0.146	0.133	None
3	1.087	0.348	0.490	0.058	None	19	3.020	0.062	0.124	0.147	None
4	3.314	0.048^*^	0.106	0.159	None	20	3.420	0.044^*^	0.105	0.163	None
5	7.621	0.002^**^	0.012^*^	0.303	a > c, b > c	21	2.115	0.136	0.211	0.108	None
6	2.976	0.064	0.124	0.145	None	22	0.244	0.785	0.811	0.014	None
7	0.997	0.379	0.490	0.054	None	23	2.649	0.085	0.146	0.131	None
8	1.023	0.370	0.490	0.055	None	24	5.908	0.006^**^	0.021^*^	0.252	a < b, a < c
9	0.747	0.481	0.552	0.041	None	25	6.172	0.005^**^	0.019^*^	0.261	a < b, a < c
10	7.822	0.002^**^	0.012^*^	0.309	a > c, b > c	26	4.433	0.019^*^	0.054	0.202	None
11	3.922	0.029^*^	0.075	0.183	None	27	2.514	0.095	0.156	0.126	None
12	0.686	0.510	0.565	0.038	None	28	10.775	0.000^***^	0.002^**^	0.381	a < b, a < c, b > c
13	0.357	0.702	0.750	0.020	None	29	13.710	0.000^***^	0.000^***^	0.439	a < b, a < c, b > c
14	6.903	0.003^**^	0.015^*^	0.283	a > c, b > c	30	14.414	0.000^***^	0.000^***^	0.452	a < b, b > c
15	1.712	0.195	0.288	0.089	None	31	5.599	0.008^**^	0.025^*^	0.242	a < b, b > c
16	0.775	0.468	0.552	0.042	None						


*a, presented with a positive facial expression picture (requiring participants to make a negative expression); b, presented with a negative facial expression picture (requiring participants to make a positive expression); c, presented with a neutral facial expression picture (requiring participant to not make any expression). PHMC, *post hoc* multiple comparisons.*

At channels 5, 10, and 14, the OxyHb concentration change for negative and positive facial expressions were significantly higher than those for neutral facial expressions. Furthermore, at channels 28, 29, 30, and 31, the OxyHb concentration change was higher for positive facial expressions than for negative or neutral facial expressions. In channels 24, 25, 28, and 29, the OxyHb concentration change was lower for negative facial expressions than for positive and neutral facial expressions.

**FIGURE 4:**
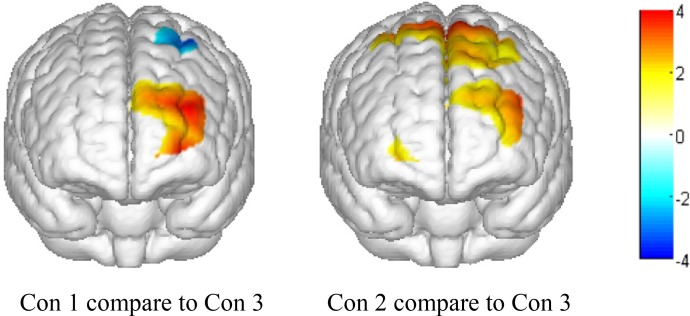
Topographical map of significant *t*-values (*P* < 0.05). Con 1, presenting a positive emotional face picture and asking participants to make a negative expression; Con 2, presenting a negative emotional face picture and asking participants to make a positive expression; and Con 3, presenting a neutral face picture and asking participants to not make any expressions.

To further visualize the OxyHb concentration change differences between positive, negative, and neutral expressions, we used the results of the main effects analysis to calculate *t*-values of the comparison between condition 1 and condition 3 and between condition 2 and condition 3. Then, we used the Topoeasy toolbox (Tian et al., 2013) of MATLAB 2013b to plot a topographical picture of the significant *t*-values (see Figure 4).

As shown in the topographical maps, compared to presenting a neutral face picture (thereby requiring participants to make no emotional expression), presenting positive and negative face pictures significantly increased OxyHb concentration in the left front and left middle areas of the PFC. Additionally, when presenting a positive face picture (thereby requiring participants to make a negative expression), the OxyHb concentration significantly decreased in the left pre-motor and supplementary motor cortex near the rear area of the PFC. Finally, when presenting a negative face picture (thereby requiring participants to make a positive expression), the OxyHb density significantly increased in the pre-motor and supplementary motor cortex near the rear area of PFC.

### Questionnaire Results

Using the Student’s *t*-test, we examined participants’ subjective feelings after completing the experiment, which could be a supplement to the results of fNIRS. The results are shown in Table 4.

**Table 4:** Status of participants after completing the experiment.

Comparison between groups *n* = 20 *n* = 18			
	**Surface acting**	**Deep acting**	
		
	***M* ± *SD***	***M* ± *SD***	***t***

Q1	1.750 ± 0.910	1.944 ± 0.938	-0.648
Q2	1.800 ± 1.005	1.889 ± 1.023	-0.270
Q3	4.100 ± 0.788	4.167 ± 1.043	-0.224
Q4	5.150 ± 0.745	5.056 ± 0.725	0.395


*Q1, Q2, Q3, and Q4 represent the four items on the questionnaire.*

For the means of all four questionnaire items, we found no significant difference between the surface acting and deep acting groups.

## Discussion

### Energy Consumption of Surface and Deep Acting

The main hypothesis of our study, which was based on action theory, is that surface acting and deep acting consume different amounts of psychological resources—particularly, deep acting leads to greater energy consumption. However, the study results appear somewhat contradictory. When we consider only the number of activated areas in the brain, deep acting activated more channels than surface acting did, thus supporting our study hypothesis. However, when looking at the ANOVA results, we observed neither significant differences in activation between surface and deep acting nor a significant interaction of the group and facial expression. In other words, these results do not support our hypothesis. Thus, when using a more strict criterion (the ANOVA results), it is possible that there is no difference in energy consumption between surface and deep acting in the PFC based on this study. However, this does not mean that we can absolutely make a conclusion: there is no difference of energy consumption between surface and deep acting. This is because in addition to the PFC, other brain regions may also be associated with emotional labor processes, such as subcortical tissue amygdala, hippocampus, and so on. In addition, in this study, we only used the instruction to ask participants to perform surface acting and deep acting, and used a video monitor to record their facial expressions. However, for the deep acting, we did not monitor whether the participants were experiencing the emotion corresponding to their facial expression (in fact it is very difficult to do). This made it difficult to confirm whether the participants really performed deep acting.

In addition to action theory, we might consider the results in light of the conservation of resources theory. According to this theory, surface acting, because it involves the suppression of emotions, consumes more resources than deep acting does (Richards and Gross, 2000), making it a strategy with greater cognitive investment (Brotheridge and Lee, 2002). However, our results do not appear to support the conservation of resources theory. We suggest three possible reasons for this. First, there might in fact be no significant difference in energy consumption between deep and surface acting (at least in the short term). This is backed by the questionnaire results: the mean score for the question “How would you rate the degree of effort put into the experiment?” was slightly higher in the deep acting group than in the surface acting group, but not to a significant degree (see Table 1). In other words, based on participants’ subjective feelings, there was no difference in deep acting and surface acting in terms of effort. Second, the fNIRS can only measure the outer cortex, surface of the brain. However, surface and deep acting may also be involved with the deeper brain structure. For this, a fNIRS study cannot discover whole brain activities related to emotional labor, and it remains difficult to verify the action theory and the conservation of resource theory. Third, emotional labor is such a complex activity that it is possible that we have not actually measured its energy consumption in this study. We simulated only the surface and deep acting strategies of emotional labor; in real emotional labor, the goals of the organization are exceedingly important factors to consider, as they will doubtlessly influence the emotional labor process (including surface and deep acting). With our design, we would have found it difficult to present participants with a definite organizational goal.

### Differences Between Making Negative and Positive Facial Expressions

We found that facial expression had a significant main effect for some channels (see Table 3). To clarify the role of emotional valence, we compared the changes in OxyHb concentration between the presentation of a positive facial expression and the presentation of a neutral facial expression, and between the presentation of a negative facial expression and a neutral facial expression. Compared with the neutral facial expression, presenting either the positive or negative facial expression (and making the corresponding opposite facial expression in response) led to a notable activation in the left front and left middle parts of the PFC (BA46 and BA10). According to the valence hypothesis, the left PFC is activated during positive emotions, whereas the right PFC is activated during negative emotions (Silberman and Weingartner, 1986; Jansari et al., 2011). Using functional magnetic resonance imaging (fMRI), Goldin et al. (2008) found that the right ventrolateral PFC (VLPFC) was significantly activated during emotional suppression while viewing disgusting images. Kreplin and Fairclough (2013), in applying fNIRS during the viewing of visual art, selected to induce positive and negative emotions and found a significantly higher increase in OxyHb in the medial rostral PFC (rPFC) when viewing positive images, as compared to viewing negative images. Furthermore, Rodrigo et al. (2016), also applying fNIRS, found that when viewing fearful as compared to neutral faces, participants demonstrated higher levels of activation within the right medial PFC. On the other hand, participants demonstrated lower levels of activation within the left medial PFC and left lateral PFC when viewing fearful faces, as compared to neutral faces. Our results differ from both these previous studies and do not support the valence hypothesis. However, according to the affective workspace hypothesis, the activity patterns of the same core neural network implement both positive and negative valences, which are determined by differences in the pattern of activation (Barrett and Bliss-Moreau, 2009; Trambaiolli et al., 2018). Lane et al. (1997a,b) presented participants with happy, sad, and disgusting film clips, and asked them to feel the relevant target emotion. They found increased activity in the left medial PFC for both positive and negative emotions, measured using PET. Honda et al. (2018) measured the change in OxyHb concentration in the PFC while participants commented on a disgusting film they had seen, and found significant differences in OxyHb concentration in the left PFC between when they engaged in emotional suppression and when they did not. Our study results are in line with the studies of Lane et al. (1997a,b) and Honda et al. (2018), and support the affective workspace hypothesis.

Additionally, compared to making no facial expressions, when participants engaged in a positive facial expression, the posterior frontal lobe (mainly the frontal eye fields and supplementary motor cortex) showed activity; however, this was not found when they made a negative facial expression. This finding might indicate that positive facial expressions are related to stronger motion control. Lane et al. (1997a) also found that happiness was distinguished from sadness by greater activity in the vicinity of the ventral medial frontal cortex. However, we cannot make any conclusions based such few studies, so further research is necessary.

### Limitations

First, in this study, we only presented participants with emotional face pictures, and asked them to display the opposite facial expression to the pictures they saw, using instructions to manipulate whether they would engage in surface or deep acting. Accordingly, our findings may not reflect real-life emotional labor. Further studies should consider motivational factors in order to improve the external validity of the study. Then, in this study, we primarily focused on PFC activity. Although the PFC is the most important zone for emotion regulation (Braunstein et al., 2017), emotional responses to visual stimuli are regulated through neural pathways in multiple brain regions, including the amygdala and PFC, and emotional labor involves other parts of the brain (e.g., the motor zone of the parietal lobe). In order to explore whether there is a difference in energy consumption between surface acting and deep acting, more areas of the brain should be monitored in the future. Finally, we did not monitor whether the participants were experiencing the emotion corresponding to their facial expression. This made it difficult to confirm whether the participants really performed a deep acting. So, in future studies, it is worth exploring how to use behavioral and physiological indicators to measure participants’ surface acting and deep acting.

## Conclusion

Based on the results of this study, we infer that deep acting and surface acting may not show a significant difference in energy consumption. Furthermore, engaging in positive and negative emotional labor may rely on some of the same psychological mechanisms, though there could be differences as well.

## Ethics Statement

All research procedures were approved by the Research Ethical Committee of Guizhou Normal University Educational School according to the Declaration of Helsinki. All participants were given written informed consent after they fully understand the study.

## Author Contributions

WW designed the experiments. YL, HZ, DL, and DP collected the data. YL and GM analyzed the data. YL, WW, and SZ wrote the main manuscript. YL and WW prepared the figures. All authors reviewed the manuscript.

## Conflict of Interest Statement

The authors declare that the research was conducted in the absence of any commercial or financial relationships that could be construed as a potential conflict of interest.
